# Biosensors in Health Care: The Milestones Achieved in Their Development towards Lab-on-Chip-Analysis

**DOI:** 10.1155/2016/3130469

**Published:** 2016-03-03

**Authors:** Suprava Patel, Rachita Nanda, Sibasish Sahoo, Eli Mohapatra

**Affiliations:** Department of Biochemistry, All India Institute of Medical Sciences, Raipur, Chhattisgarh 492099, India

## Abstract

Immense potentiality of biosensors in medical diagnostics has driven scientists in evolution of biosensor technologies and innovating newer tools in time. The cornerstone of the popularity of biosensors in sensing wide range of biomolecules in medical diagnostics is due to their simplicity in operation, higher sensitivity, ability to perform multiplex analysis, and capability to be integrated with different function by the same chip. There remains a huge challenge to meet the demands of performance and yield to its simplicity and affordability. Ultimate goal stands for providing point-of-care testing facility to the remote areas worldwide, particularly the developing countries. It entails continuous development in technology towards multiplexing ability, fabrication, and miniaturization of biosensor devices so that they can provide lab-on-chip-analysis systems to the community.

## 1. Introduction

Since the development of the first oxygen biosensor by Led and Clark in 1962, biosensors have gained enormous attention in recent years in medicine and nanotechnology. The biosensor products have shown an immense potential for applications in medical diagnostics and numerous industries like pharmaceutical, food, beverages, environmental, agricultural, and many other biotechnological industries [1]. Because of the high demand in the market, blood glucose monitoring is the major application of biosensors so far. The biosensor products have been successful in achieving very high level of precision in measuring disease specific biomarkers not only in* in vitro *environment,but in* in vivo* environment as well [2]. The biosensing components used in biosensors are highly capable of sensing the real time signals such as production of biomolecules like glucose, lactate, peroxides, and cytokines and release of proteins or antibodies in different inflammatory diseases and tumors. These biosensors can efficiently detect the target molecule in very low quantities and are considered to be powerful tool to detect disease at its initial stage and start the treatment early [3]. The above unique advantage of biosensors has encouraged researchers to develop more and more newer technologies and the industry is now worth billions of dollars.

Recently, researchers have come up with various innovative strategies to miniaturize these devices so that they can be used as an active integral part of tissue engineering systems and implanted* in vivo* [4, 5]. These devices have ultrasensitive sensing systems to precisely perceive the changes in biological signals in a cellular microenvironment. Surface plasmon resonance (SPR) [6], nanotubes, nanowires [7, 8], or nanocantilevers [9] are used for this purpose to quantify very low levels of biomolecules including specific DNA moieties. Quantum dots are another group of innovations which are highly fluorescent semiconductor nanocrystals and use the principle of fluorescence resonance energy transfer (FRET) for signal transduction [10]. Researchers are in progress to synthesize nanobiosensors that are biocompatible and have enhanced signaling potential, to be delivered along with therapeutic delivery devices for* in vivo* screening and treatment.

Lower detection limits, high level precision and accuracy, high specificity, ultrasensitivity, fast and simple assay techniques, very low reagent consumption, and many biological sensing elements are reusable and allowable to configure the device for continuous monitoring or automatic process control to optimize the measurement of some crucial parameters that are the advantages that could be exploited for replacing time consuming laboratory analyses in medical diagnostics towards bedside point-of-care testing.

This paper reviews the recent innovations on biosensors and their prospective/potential applications in medical diagnostics.

## 2. Innovations of Biosensors

**Figure 1:**
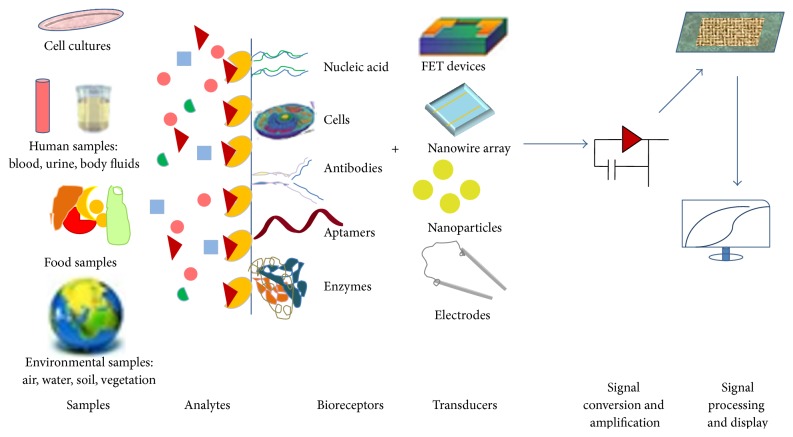
Schematic diagram showing the components of a biosensor. Reproduced after editing from Grieshaber [12].

A biosensor is defined as “a self-contained analytical device that combines a biological element (biosensing components) with a physicochemical component (biotransducer component) to generate a measurable signal for detection of an analyte of biological importance.” It consists of three basic components: (i) a detector to detect the biomolecule and generate stimulus, (ii) a transducer to convert the stimulus to output signal, and (iii) a signal processing system to process the output and present it in an appropriate form [11] (Figure 1).

## 3. Biosensing Elements

Biosensing elements are a set of biological entity, those that are capable of carrying out specific group reactions or can bind with particular group of compounds, to yield a detectable signal that is read and transformed by the transducers. Commonly used biosensing elements are of two types, namely, catalytic type and affinity type. The catalytic sensors include enzymes, microbes, organelles, cells, or tissues. The affinity type sensors are antibodies, receptors, and nucleic acids [15].

### 3.1. Enzymes

Enzymes like glucose oxidase (GOx), horseradish peroxidase, and alkaline phosphatase have been widely used in many biosensor studies. The enzyme based biosensors utilize the principle of enzyme catalytic reactions accompanied by consumption or generation of detectable compounds like O_2_, CO_2_, H_2_O_2_, NH_3_, and H^+^ or by activation or inhibition of the enzyme activity by the analyte that can be easily detected by the transducers. These biocatalysts can be directly immobilized on the transducers by gel entrapment technology, covalent bonding, or physical adsorption. Enzyme based biosensors have been extensively studied because of their medical applicability, commercial availability, and ease of enzyme isolation and purification from different sources [16]. The major advantage of using enzymes as biorecognition element is their aptness for modification of active sites by genetic engineering and thus modifying their substrate specificity to detect a wide range of analytes. Besides, the catalytic action of enzymes remains unaltered till the end of the reaction; the sensors can be used continuously. The limitations of these enzyme based biosensors are due to the limited enzyme stability and dependency of their activities on factors like pH, ionic strength, chemical inhibition, and temperature. Though* de novo* designing modifies the enzyme substrate specificity, at the same time it jeopardizes its kinetic property and reaction rate [17]. Recent articles have updated various new strategies for making use of enzyme stabilization in enzyme based biosensors. Carbon nanotubes (CNTs) due to their excellent electroconductivity and tensile strength are very much suitable to act as a scaffold for enzyme immobilization and enhance electron transfer to the electrodes [18, 19]. The tendency of CNTs deposition along the electrode surface and forming a jumbled meshwork limits their usefulness in this technology. However, new approaches of integrating the CNT surfaces with biopolymers or using dissolved CNTs in a mixed solution of cyclodextrin and its prepolymers can maintain the bioactivity of the immobilized enzymes on it for a longer time. Such chemical modification can be utilized for fabricating more stable chemically modified electrode surfaces [20]. Further modifications of CNTs have been successfully achieved in improved sensitivity by tailoring the thickness of scaffolds [21], covalent immobilization of organophosphorus hydrolase (OPH) enzymes [22], or covalent modification of glucose oxidase (GOx) on carboxy-functionalized grapheme sheets [23] or grapheme-chitosan nanocomposite films [24]. Besides CNTs, sol gels/hydrogels have been extensively used for providing an excellent conducive base for enzyme immobilization in constructing the third-generation enzyme based biosensors. These matrices are fabricated using metal oxide preparations such as silica-encapsulated OPH [25] and gold nanoparticles (AuNPs) embedded with horseradish peroxidase (HRP) [26] or GOx that immensely increase the sensitivity of detection range of blood glucose by the biosensor in a linear range of 0.1 to 10 mM [27]. The immobilization of enzymes and their alignment on electrode surfaces can be modified by constructing apoenzymes that need a specific cofactor to function. Apoenzymes can be reconstituted and linked to cofactor functionalized nanostructures on the electron conducive area. Apo-GOx coupled to AuNPs integrated with cofactor flavin adenine dinucleotide (FAD) shows an enhanced electrical conductivity on the electrode surface [28].

### 3.2. Microbes

Microbes have been used as biosensing matrix in fabrication of biosensors. Their major advantages are that they are present ubiquitously, adapt to undesirable environment, and are capable of metabolizing new molecules with time. When compared to enzymes, whole cell microbial biosensors are more economical and capable of metabolizing complex compounds either aerobically or anaerobically releasing various molecules like ammonia, carbon dioxide, hydrogen ions, and so forth; those can be monitored by different transducers. Unlike enzymes, microbial biosensors do not require purification step which is again time consuming and expensive. Because of the unique advantage of microbes to detect the bioavailable fraction of the contaminant over the total concentration, these biosensors are used widely for environmental monitoring like pollutants or pathogens in air, water, soil, or food and assessing biological oxygen demand in wastewater. The major disadvantage is the limited understanding of the biochemistry involved and difficulty to transpose the information gathered through microbial whole cell sensors and apply it directly to higher organisms. Other limitations include their short lifetime, unreliable operation in complex environment, low signal-to-noise ratio, and lack of genetic stability, which leads to variability in the response of different cells. As in enzymes, procedural conditions like pH, temperature, incubation time, and reagents can also affect the performance of the biosensors [11, 29, 30]. Microorganisms have been exploited for clinical diagnosis of hormones, pathogens, toxins, and other analytes. An* E. coli *SOS-EGFP based on SOS response was constructed for detection of DNA damage [31].

### 3.3. Organelles

Each organelle carries out specific function inside a cell and hence can be utilized in biosensing the specific analyte. For example, mitochondria can measure calcium concentration because of their ability to concentrate calcium in them. This ability is used to detect the water pollutants [32].

### 3.4. Cells and Tissues

Cells have the ability to modify as per the surrounding environment for which they are subjected to be used as biosensing component. Adhesiveness to surface is another characteristic advantage that makes it a suitable candidate for immobilization on the matrix surface and attachment of receptors on cell membrane. They are often used in monitoring treatment effects of drugs, toxin levels, level of different stress factors, and organic derivatives. Tissues are advantageous over cells and organelles because of high content of enzymes, cofactors, higher activity, and stability. But they lack specificity because of presence of unwanted enzymes which leads to ambiguous catalytic reactions [15, 33]. Single-cell analysis of neuronal cells during neuronal regeneration can be achieved by quantitative measurement of cellular transmitter released by the cells trapped in a closed microchip close to a band of microelectrodes [34]. Cell based microfluidic technology is most suitable for cell migration assay and invasion assay applicable in drug screening. It can quantify the migrating cells in response to chemotactic gradient across a physical barrier [35]. Breast cancer cell detection at single-cell resolution was achieved using high density electrochemical impedance biosensor array for tumor cell detection [36].

### 3.5. Antibodies

The antibody is a critical part of immunosensors. These immunosensors utilize the principle of highly selective antigen-antibody reaction. The antibodies are immobilized on the surface of matrix in an array format and linked to the transducers covalently through amide, esters, or thiol. The antibodies interact with the analyte, allowing modification at the functional groups attached to transducer surface for detection and quantification. They are more specific and faster as compared to other traditional immunoassays like ELISA test. These are widely used for infectious disease diagnosis [37]. There are also some limitations such that antigen-antibody complex formed is irreversible and so a single array can be used only once. Antigen-antibody interaction also depends on the strength of affinity and the orientation of the antibody when it is immobilized on the surface.

### 3.6. Nucleic Acids

DNA is an appropriate candidate for biosensing because of its specific ability of base pairing with complementary sequence. Nucleic acid biosensors (NABs) employ short synthetic single-stranded oligonucleotide probe that is immobilized on the transducer to detect the DNA/RNA in the sample [38]. These probes can be reused because the hybridization between probes to the sample can be denatured to reverse binding and then regenerated. But the limitation lies in the sample DNA quantity because, for accuracy of the result, the sample DNA content has to be multiplied to readable quantity by polymerase chain reaction (PCR) which is again time consuming. Researchers are working on developing biosensing elements to identify the natural DNA/RNA from the organism and in human blood with a view towards a successful application for point-of-care testing of metabolic disorders (diabetes, cardiovascular diseases), infectious diseases (tuberculosis, hepatitis, dengue, cholera, and salmonellosis), cancer, and genetic diseases [39]. At present, microRNA (miRNA) based biosensors act as an ultrasensitive tool to detect cancer associated miRNAs in serum sample [40].

### 3.7. New Receptors: Aptamers

Aptamers are regarded as a new frontier. These are artificial single-stranded DNA or RNA ligands that can be generated against amino acids, drugs, proteins, and other molecules. The advantage is that the oligonucleotides attain a stable secondary structure that can be easily synthesized and functionalized. The aptamers bind to the target with selective affinity and efficiently discriminate between closely related targets [41]. For their appealing features, aptamers are selected as therapeutic agents and, for the first time, an aptamer has recently been approved by US Food and Drug Administration for the clinical treatment of age related ocular vascular disease [42]. The application in diagnostic field is still under investigation and needs further advanced research.

## 4. Biotransducer Elements

Transducers are the elements which identify the stimulus released from the interaction of the analyte with the biosensing component and transform it into a detectable signal. Of all the developed biosensors, the commonly used are electrochemical, optical, and piezoelectrical [60].

### 4.1. Electrochemical Sensors

**Figure 2:**
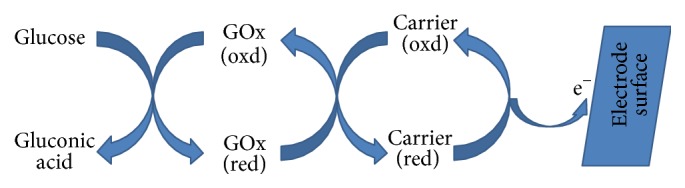
Diagrammatic representation of an enzyme modified electrochemical biosensor.

These transducers measure the electrochemical changes that occur on the sensing surface of electrodes on interacting with the analyte (Figure 2). As per the electrical changes, it can be a potentiometer (a change in measured voltage), amperometric (a change in measured current at a certain voltage), and conductometric (a change in the ability of the sensing material to transport charge). The advantages of these biosensors are that they are simple and cost effective because of use of electrodes and can be easily miniaturized towards fabrication of implantable biosensor. This technique is used commercially for detection of DNA/RNA, enzyme based assays like glucose and in field monitoring (e.g., handheld) [61].

In electrochemical NABs, DNA is embedded onto the electrode surface and change in electrical conductance is measured after the hybridization reaction. Label based indirect assay utilizes the principle of sandwich method where the analyte is sandwiched between the capture and detector agents. The capture agents such as heterocyclic dyes, ferrocene derivatives, and organometallic complexes are usually immobilized on electrodes, glass chips, and nano- and microparticles. The detector agents are typically conjugated to signaling tags like fluorophores, enzymes, or nanoparticles (NPs) [39, 62]. This method is utilized for detection of proteins, peptides, antibodies, and nucleic acids. The best commercially available sandwich assays are lateral flow immunoassays or immunochromatographic test strips, for example, home pregnancy tests and urinalysis strips. The signals can be measured qualitatively visually or also semiquantitatively by photodiode or amperometric detectors [63]. Label-free biosensors determine the changes when the target analyte binds to the capturing agent immobilized on the solid support. The advantages of label-free detection are that it requires only one recognition element, reduced analysis time, and low reagent cost. It allows continuous data monitoring and real time analysis. The analytes are estimated in their natural form without any chemical alteration [62, 64]. A unique label-free DNA biosensor recently introduced as metal film on nanosphere (MFON) is based on Molecular Sentinel (MS) immobilized on a plasmonic “nanowave” chip. It utilizes the principle of reduced surface-enhanced Raman scattering (SERS) intensity occurring due to DNA hybridization. The potential application of this biosensor is to detect human radical S-adenosyl methionine domain containing 2 (RSAD2) gene which is a common inflammation biomarker [65]. Label-free analysis of proteins includes the aggregate proteins of neurodegenerative diseases like Parkinson's disease [66], Alzheimer's disease [67], and tumor suppressor protein p53 [68] and analysis of poorly soluble membrane proteins like sodium potassium ATPase [69].

### 4.2. Optical Sensors

**Figure 3:**
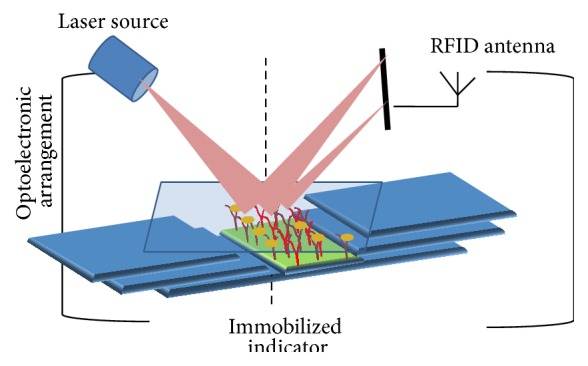
Architecture of an optical biosensor. Reproduced from Dey and Goswami [13].

The output transduced signal that is measured in these sensors is light based on its optical diffraction. Light in an optical device is directed towards the sensing surface through optical fibers or interferometer or dielectric waveguides and reflected back again (Figure 3). The reflected light is screened by a detector such as photodiode that calculates the physical changes occurring on the sensing surfaces. These biosensors are particularly applied for detection of bacterial pathogen and for studying the kinetics of antigen-antibody and DNA interactions. These sensors can perceive the microscopic changes in refractive index or thickness when cells interact with the immobilized receptors on the transducer surface. The change in the properties of light correlates with the changes in mass, concentration, or number of molecules in the cell. The measured optical signals often include absorbance, fluorescence, chemiluminescence, surface plasma resonance, or changes in light reflectivity [61, 70, 71]. They are preferable biosensors for screening a population of samples simultaneously. The drawback of these systems is that they cannot be miniaturized easily and require a spectrophotometer to measure the signals.

### 4.3. Piezoelectric Sensors

Piezoelectric sensors are also called mass sensors; the working principle of these biosensors is based on the interaction regarding the amount of analyte with the sensing element, usually a vibrating piezoelectric (PZ) quartz crystal. When an analyte of interest binds to the PZ sensing element, the resonant frequency of the PZ crystal changes. This creates an oscillating voltage that is spotted by the acoustic wave sensor. Widest use of these sensors has been in gas phase analyses. These sensors have also the same limitations like that of optical sensors that also require sophisticated instruments and are not easy to be miniaturized.

### 4.4. Thermal or Calorimetric Sensors

These types of biosensors take advantage of the fundamental properties of a reaction, that is, adsorption and heat generation. As a result of biological reaction, the temperature of the medium changes; this is measured and compared to a sensor with no reaction to determine the analyte concentration. These biosensors are most suitable for enzyme based reactions. They are commonly used for estimating pesticides and pathogen bacteria but also used to measure serum cholesterol based on enzymatically produced heat of oxidation and decomposition reaction.

There are also many other biosensors that exploit the principle of acoustics, magnetism, and bioluminescence which are not very widely accepted for clinical diagnostic applications.

## 5. Newer Generation Biosensors: Nanobiosensors

### 5.1. Quantum Dots

Sensitivity and specificity of optical biosensors can be enhanced if coupled to quantum dots (QDs). QDs are nanometer-scale semiconductor crystals with unique quantum confinement effects. They have a broad excitation and narrow size-tunable emission band width, negligible photobleaching, and ultrastability [1, 72]. They work on the principle of fluorescence transduction due to direct or indirect interaction of analyte with the QD surface, either through photoluminescent activation or through quenching. Surface alterations (carboxy-functionalized) of the QDs have started the development of multimodal probe based biosensors that can directly link with the targeted peptides, nucleic acids, or ligands. These nanocrystals have a wide variety of applications ranging from detection of pH and ion to quantification of organic derivatives and biomolecules (DNA, RNA, enzymes, proteins, amino acids, and drugs). Applications are hindered because of their known high toxicity and limited reusability [73]. Further advancement is required in synthesis process and conjugation methods in order to overcome the challenges.

### 5.2. Graphene Based Biosensors

Graphene is a sheet of densely organized carbon atoms in honeycomb (hexagonal) pattern. The 2D structure of graphene provides a large surface area and excellent electrical conductivity to allow it to act as a conductor of electrons between the redox centers of proteins or enzymes and the electrode's surface. Rapid electron transfer enables accurate and selective detection of biomolecules. They are advantageous over carbon nanotubes in terms of low cost, large specific surface area, good compatibility, and better electrocatalytic performance. They possess less of contaminants like transition metals Fe, Ni, and so forth, thus considered to be more pure than the carbon nanotubes, and thus provide better platform to study electrocatalytic activity of carbon atoms and better understanding of nanostructures in general which indirectly will be applied in advancement of nanotechnology. For its high tensile strength and other characteristics, graphene is now a preferred choice for the fabrication of various biosensor devices.

Graphene based electrodes are used for detection of small molecules like H_2_O_2_, NADH, glucose, amino acids, and neurotransmitters. These electrodes employ the principle of oxidation-reduction reaction on their surfaces. The grapheme electrodes are modified (chemically reduced grapheme oxides or multilayer nanoflake film), in order to increase the electron transfer rate compared to the other electrodes, contributing to high biosensing performance [74, 75].

Graphene can also be excellent electrode material for electrochemical biosensors. Graphene based enzyme biosensor like glucose biosensors can be used in regenerative medicine for continuous monitoring of metabolic activities. The enzymes like glucose oxidase are linked covalently and immobilized to the chemically modified graphene. Graphene based nanocomposite materials are also used to assess the biomolecules; for example, graphene decorated with gold nanoparticles/Nafion nanocomposites biosensors shows a very fast response in detecting glucose molecule as well as environmental contaminants like heavy metal ions. These are nonenzymatic biosensors that have high sensitivity and long-term stability. Graphene based electrochemical DNA biosensor offers high sensitivity and selectivity for detection of specific DNA sequence or mutated genes in a particular human disease.

**Figure 4:**
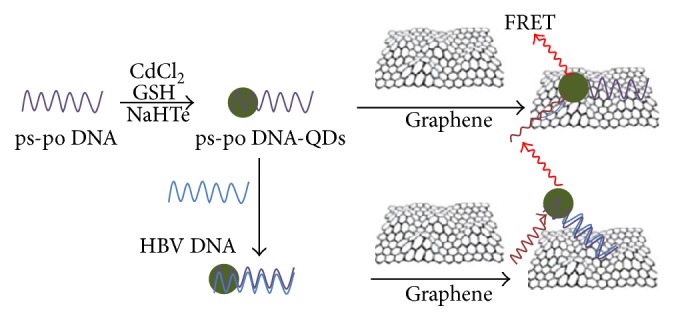
Graphene based biosensing platforms reproduced from Pineda et al. [14] (http://researchgate.net/). Schematic for the preparation of DNA-CdTe quantum dots (QDs) for a FRET assay of DNAs.

Graphene quantum dots (Gdots) based biosensors, such as 0D Gdots, are photoluminescent materials derived from graphene or carbon fibres. They too possess the unique optical properties of quantum confinement and a wide range of excitation-emission spectra. The Gdots are superior to other imaging agents like cadmium Qdots due to their higher photostability against photobleaching, better biocompatibility, and low toxicity. These features enable the Gdots to be coupled in electronic sensors and electrochemical and photoluminescence sensors (Figure 4). The tunable size of Gdots permits analyses of ssDNA, enzyme immobilization, and avian leukosis virus. Gdot based electrochemiluminescence sensor also allows detection of metal ions and amino acids. The planar surface of Gdots when modified with gold nanoparticles enhances the detection limit to very minute levels [76, 77].

### 5.3. Carbon Nanotubes

Carbon nanotubes (CNTs) are cylindrical fabrication of rolled-up graphene sheet. CNTs based biosensors are promising candidates for biomedical application because of their attractive chemical and physical properties derived from graphene. Because of the strength of atomic bonds in carbon nanotubes, they can withstand very high temperature and act as excellent thermal and electrical conductor. Antibodies or specific probes coated on these nanotubes can detect the antigens like viruses, nucleic acid, enzymes, and biomolecules. The CNT based biosensors operate on the principle of change in electrical conductivity correlating with the distance between the target analyte and the probe which is readable by the electrical meter. The CNTs can also be paired with electrochemical biosensors to enhance the sensitivity of the enzyme electrodes, immunosensors, and nucleic acid biosensing. Because of their amazing tensile strength and elastic behavior, they can be easily twisted, pliable, and miniaturized. The main disadvantage is the synthesis of pure form of CNT without losing much of its properties. Besides three barriers in terms of functionalization, pharmacology and toxicity of CNTs limit their extensive application in biomedicine. They possess limited solubility in aqueous medium and their pharmacokinetics depends on their shape, size, chemical composition, and aggregation ability which is not yet cogent. These nanoparticles being under 100 nm can easily escape phagocytosis and inflammatory response and can endure redistribution from its original site. CNTs have been widely investigated for promising application in oligonucleotide and enzyme based sensors. CNTs are unique in the sense that both the advantages and limitation can be exploited for biomedical application. High elasticity and tensile strength make it possible to act as bone implant or implant substitute along with calcium chips, into the bone structure, whereas, because of the ultra-small size and defense escaping property, it can be employed as implant in artificial joint without host rejection response. Due to nanosize of CNTs, they can efficiently enter the cells and organelles to interact with the proteins overexpressed in cancer cells at the very initial stage of cancer. The ultrahigh surface area makes it a novel agent for delivery of drugs, peptides, and nucleic acids [78, 79].

### 5.4. Microfluidic Biosensors

These are considered as analytical devices in which the biologically active component (receptor) is immobilized onto the surface of an electronic transducer allowing the detection of target analyte in a viscous liquid medium. The sensing technology recognizes the change of mass on the surface or change of dielectric behavior in the presence of tumor marker or pathogen. The devices are characterized by high surface-area-to-volume ratios. In these fluidic systems, the flow current may be pressure driven, electrokinetic based, or based on electroosmosis. The system can be employed with electrochemical, mechanical, and optical transduction technologies. The microfluidic platform allows handling of very tiny volumes of expensive reagents, enables detection of target molecules in increasingly smaller concentration (down to 0.2 fM), and permits integration of several functions. The multiplexing ability along with lower detection limit has been lucrative idea for fabrication of these systems for point-of-care (POC) applications. In regenerative medicine also, the microfluidic scheme provides excellent evaluation of biomolecules participating in the functionality of the engineered tissue. An efficient microfluidic cell culture system allows precise control of cellular metabolism, cell adhesion, monitoring of cellular metabolites, and mimic signals that direct cell fate to create specific organ construct. Pairing POC facility with microfluidic design is a key challenge for researches in regenerative medicine, as many biomarkers have to be monitored to evaluate the functionality of any tissue engineered construct* in vitro*. There are still challenges in developing integrated functioning device that provides real clinical application value. Development of such integrated devices needs extensive miniaturization of the pumping system which is extremely complicated and expensive. It is very difficult to know the actual transport of molecules through the system and thus there is lack of sufficient data relating to their testing ability with complex sample specimens [80–82].

### 5.5. *Lab-on-a-Chip*

A miniaturized device of utmost diagnostic importance integrates onto a single chip capable of analyzing one or several parameters including biomolecules, DNA, or RNA. The main technology that applies to development of* lab-on-a-chip* is microfluidics and molecular biotechnology. These devices are fabricated with numerous microchannels embedded with antibodies, antigens, or oligonucleotides, enabling thousands of biochemical reactions from a single drop of blood. Commonly, polydimethylsiloxane (PDMS), thermoplastic polymers, glass, silicon, or paper based technologies are employed for fabrication of* lab-on-a-chip*. However, PDMS and paper based* lab-on-a-chip *are more widely used because of their low cost and being easy to fabricate.

- Applications in molecular biology:* lab-on-a-chip *allows fastest way of PCR by performing high speed microscale thermal shifts. It can incorporate an array of DNA to bring about thousand times faster genome sequencing.
- Applications in proteomics: the device has a great potential to integrate all steps of proteomics starting from extraction, separation, electrophoresis, analysis using mass spectroscopy, and crystallization of proteins.
- Applications in cell biology: it can deal with large number of cells in seconds. Nonetheless, it has the ability to control cells at single-cell level simultaneously. It can detect, isolate, and sort out a single specified cell when programmed.

The advantages of* lab-on-a-chip *are its low cost, comparable sensitivity to conventional diagnostic methods, rapid testing time, ease of use, being handy to carry due to its compactness, low volume samples, and real time monitoring; moreover it can be used anywhere without any environmental interferences.

In Table 1 are given some new innovations and updates of recently developed biosensor technology for measuring various analytes.

## 6. Conclusion

Since the invention of Clark's electrode in 1950s, enormous development has been achieved in the field of biosensor technologies in these sixty-five years. However, the practical application of biosensors in medical world is still in its infancy. In order to meet the criterion of a precise diagnostic tool, these devices need further advancement in terms of simplicity, sensitivity, multiplex analysis of multiple biomarkers, and integration of different functions by the same chip. The electrochemical and optic based biosensors are firmly established in clinical chemistry laboratories routinely for evaluation of blood parameters like glucose, lactate, urea, and creatinine and also POC testing of glucose. Immunosensors lack popularity due to their sensitivity issues for many biomarkers when compared to the conventional immunoassay methods. However, they depict high sensitivity and faster analysis in near-patient testing for cardiac and few cancer markers. In present era, major focus is on cancer related clinical testing with improved ease of use and faster error-free analysis of tumor markers. The aim of such research is directed towards development of biosensing tools for molecular testing at the community health settings and underserved population. It necessitates continuous development and validation of biomarkers, development of ligands for those biomarkers, sample preparation methods, and multiplexing ability to analyze many cancer markers simultaneously. Besides biomarkers, exploring the genetic signatures of the tumor profile has opened new opportunities for utilizing biosensors in cancer testing. Sensitivity of DNA biosensors in targeting a single molecule in the direct sample is the chief goal to be attained. POC molecular testing requires ultrasensitive transducer technology, interchangeable biorecognition elements, miniaturization, integration, and automation of technology in order to replace sample preparation and amplification steps, reduce sample and reagent volume, and complete validation of the device in clinical testing. Development of a biosensor with the above-mentioned features is the major limitation for the rapid growth of these technologies at a competitive cost. Nanotechnology and lab-on-chip-analysis systems are the potential technologies that are capable of providing homogenous sensing format, microfabrication, and real time monitoring of the biomolecules. However, the cost needs to be adjusted in such a way that it can be affordable for all groups of people without compromising the quality control. It requires a concerted multidisciplinary approach for the development of clinically useful biosensor in the market at a reasonable price.

## Figures and Tables

**Table 1:** Recently developed biosensors along with their principles of working and applications.

Sl. number	Analyte	Biorecognition element	Sample	Technology	Advantages	References
1	Ebola, dengue, and yellow fever virus	Antibody tagged multicolored silver nanoparticles onto small strip of paper	Blood	Paper strip based multiplex disease diagnostics	Detecting down to tens of ng/mL	[43]

2	Ebola virus glycoprotein	Fe_3_O_4_ magnetic nanoparticle (nanozyme)	Blood	Nanozyme strip	Lower detection limit: 1 ng/mL	[44]

3	Urinary pathogens like *E. coli* and *Enterococcus faecalis*	Glass-polymer hybrid chip forms a centrifugal microfluidic platform that captures bacteria directly	Urine sample	Microfluidics and Raman microscopy	Detection within 70 minutes	[45]

4	*Candida* infection	Nanoparticles with supermagnetic properties coated with target-specific binding agents	Blood	Miniaturized magnetic resonance that measures water molecules reaction in the presence of magnetic fields	91.1% sensitivity, 99.4% specificity, and 1 CFU/mL (colony forming unit per milliliter)	[46]

5	Circulating tumor cells (CTC): metastatic breast, prostate, and melanoma cancers	The microchip allows microfluidic path in many rows through which the blood is pushed through	Blood	Microfluidic chip called cluster chip. CTC clusters are isolated through specialized bifurcating rows under low pulling forces preserving their integrity	At blood flow rate of 2.5 mL/hr, chip captured 99% of four- or more cell clusters, 70% of three-cell clusters, and 41% of two-cell clusters	[47]

6	Antibiotic sensitivity of bacteria	Series of minute flow-through wells patterned onto a glass chip. Each microwell coated with microbeads to trap the bacteria with the antibiotic and the signal molecule resazurin	Bacterial culture	Electrochemical reduction signal brought by the metabolism of resazurin in resistant bacteria, detected by the electrodes built on the chip	Bacterial resistance profile available within an hour of incubation	[48]

7	Ebola virus: glycoprotein (GP), nucleoprotein (NP), and viral matrix protein (VP40)	3 mouse monoclonal antibodies against each of the proteins	Blood	Chromatographic/lateral flow immunoassay SD biosensor product used for WHO EUAL Program (Emergency Use Assessment and Listing)	Sensitivity, 84.9% (95% CI ) (78.6–91.2); specificity, 99.7% (95% CI) (99.1–100.0)	[49]

8	Blood glucose (noninvasive)	Nanoengineered silica glass with ions that fluoresce in infrared light when a low power laser light hits them	Skin touch to the glass (no finger prick required)	Low-powered lasers penetrate the skin and measure the length of time the fluorescence remains and calculate the blood glucose	Wearable, noninvasive device	[50]

9	*Mycobacterium tuberculosis*	Surface modified cadmium-telluride quantum dots, gold nanoparticles, and two specific oligonucleotides against early secretory antigenic target 6	Sputum	Sandwich form FRET based biosensor to detect *M. tuberculosis* complex and differentiate it from *M. bovis* bacillus Calmette-Guerin (present in vaccinated individuals)	94.2% sensitivity and 86.6% specificity, 10-fold lower detection limit	[51]

10	*Microcystis* spp. (MYC)	Sequence-selective DNA probe to MYC and redox surface modified with ionic liquid and pencil graphite electrode	Biological sample	Electrochemical DNA biosensor using differential pulse voltammetry (DPV)	Lower detection limit: 3.72 *µ*g/mL	[52]

11	Human epidermal growth factor receptor 2 (HER2) protein in breast cancer cells	Anti-HER2 immobilized to nanoconducting film tagged to a biconjugate of hydrazine-gold nanoparticle aptamer	Blood and other body fluids	Electrochemical nanobiosensor where hydrazine acts as electrocatalyst and aptamer as reporter molecule	Ultrasensitive detection limit up to 26 cells/mL of human serum sample	[53]

12	Acoustofluidic sputum liquefier	Micromixer using oscillating sharp edges	Sputum	Microfluidic-based on-chip liquefaction device	Liquefying sputum samples at a throughput of 30 *µ*L/min	[54]

13	Pathological biomarkers	The bacterial cell acts as a diagnostic agent by inserting a “transcriptor,” equivalent of a computer program into its DNA. The bacteria act as sensor modules to detect disease signals (molecular signals that control gene expression) in clinical samples	Blood and urine	Whole cell biosensors called “bactosensors.” They are genetically encoded digital amplifying genetic switches that perform signal digitization and amplification, multiplexed signal processing with the use of Boolean logic gates, and data storage	Transcriptor amplification ability could be used to detect very small amount of biomolecules in biological samples; for example, the transcriptor connected to bacterial system that responds to glucose could detect pathological glycosuria in diabetic patients	[55]

14	Urogenital schistosomiasis	Sensor surface modifies with oligonucleotide probes targeting the 16S rRNA of uropathogen. Cells in samples are lysed, tagged with detector probe and layered sensor surface	Urine	Electrochemical biosensor composed of three gold electrodes suitably modified with capture probes. Enzyme tag mediates an amperometric signal output proportional to the quantity of the target	Can detect the presence of pathogen in an hour	[56]

15	Cervical cancer	Microbeads coated with anti-EpCAM (epithelial cell adhesion molecule), anti-CD44, and anti-TACD@/Trop2 (tumor associated calcium signal transducer 2)	Abnormal PAP smear	Smartphone imaging system called D3 (digital diffraction diagnosis). It consists of battery-powered LED light with high resolution imaging data with camera	Capable of imaging 10 megabytes of data in 0.09 secs The D3 system can categorize the biopsy samples as high risk, low risk, or benign comparable to that of conventional histology	[57]

16	Human IgG in early prostate cancer	Citrate ligands-capped gold nanoparticles are mixed with blood sera forming a protein corona around the nanoparticle surface	Blood	Gold nanoparticle enabled dynamic light scattering assay (NanoDLSay)	It shows 90–95% specificity and 50% sensitivity	[58]

17	Molecular markers	Antibody against the specific biomarker	Biological samples	Optical fluorescence spectroscopy (OFS). It consists of microfluidic channel embedded with interdigitated microelectrodes array, analyte manipulation system, and novel amplification strategy for the binding signal and highly sensitive CMOS phototransistor (complementary metal oxide semiconductor)	It is a “multi-labs-on-chip” that shows sensitivity for ultra-low level in attomolar (10–18 M) concentration of biomarkers	[59]
